# The switch‐liker's guide to plant synthetic gene circuits

**DOI:** 10.1111/tpj.70090

**Published:** 2025-03-07

**Authors:** James P. B. Lloyd, Adil Khan, Ryan Lister

**Affiliations:** ^1^ ARC Centre of Excellence in Plants for Space, School of Molecular Sciences The University of Western Australia Perth Australia; ^2^ Harry Perkins Institute of Medical Research The University of Western Australia Perth Western Australia Australia

**Keywords:** synthetic biology, gene circuits, biotechnology, genetics

## Abstract

Synthetic gene circuits offer powerful new approaches for engineering plant traits by enabling precise control over gene expression through programmable logical operations. Unlike simple ‘always‐on’ transgenes, circuits can integrate multiple input signals to achieve sophisticated spatiotemporal regulation of target genes while minimising interference with host cellular processes. Recent advances have demonstrated several platforms for building plant gene circuits, including systems based on bacterial transcription factors, site‐specific recombinases and CRISPR/Cas components. These diverse molecular tools allow the construction of circuits that perform Boolean logic operations to control transgene expression or modulate endogenous pathways. However, implementing synthetic gene circuits in plants faces unique challenges, including long generation times that slow design‐build‐test cycles, limited availability of characterised genetic parts across species and technical hurdles in stable transformation. This review examines the core principles and components of plant synthetic gene circuits, including sensors, integrators, and actuators. We discuss recent technological developments, key challenges in circuit design and implementation, and strategies to overcome them. Finally, we explore the future applications of synthetic gene circuits in agriculture and basic research, from engineering stress resistance to enabling controlled bioproduction of valuable compounds. As this technology matures, synthetic gene circuits have the potential to enable sophisticated new plant traits that respond dynamically to environmental and developmental cues.

## Significance Statement

Synthetic gene circuits have the potential to revolutionise how plants can be engineered by allowing highly customised gene expression patterns to be designed. Gene circuits can sense and integrate multiple signals and then alter plant growth to benefit basic understanding in plant sciences, or to engineer novel traits into crops.

## INTRODUCTION

A first generation of genetically modified (GM) crops with simple ‘always‐on’ traits exists, with herbicide resistance or insecticide properties dominating the market (High et al., 2004; Raman, 2017). These plants are examples in which the expression of a transgene does not impose a significant stress response, sometimes referred to as metabolic burden in synthetic biology communities (Snoeck et al., 2024), which enables the plants to stay healthy and maintain productivity while benefiting from the engineered trait. However, many traits conferring new functions to plants via an always‐on transgene come at a high cost, interfering with host cell functions to an extent that prohibits their use, for example with oil production in leaves (Vanhercke et al., 2017) and expression of pathogen resistance genes (Derbyshire et al., 2024; Gurr & Rushton, 2005). Consequently, there is a need for more sophisticated approaches to control the expression of transgenes to reduce the burden on the plant without losing the benefits of the trait (Cuzick et al., 2009; Jeong & Jung, 2015; Kidd et al., 2011; Nakashima et al., 2007; Su & Wu, 2004; Thatcher et al., 2009). The use of tissue‐ and condition‐specific promoters could reduce these incompatibilities (Aoyama & Chua, 1997; Zuo et al., 2000). However, the use of such promoters is often limited by the expression levels they confer, which may be insufficient to appropriately achieve the desired trait. Furthermore, single tissue‐ or condition‐specific promoters may not adequately define the desired spatiotemporal expression pattern for a trait, and there has been limited characterisation of natural plant promoters in diverse species to date. Synthetic gene circuits have the capability to overcome many of these challenges by enabling precise and customisable expression of desired traits by integrating multiple desired promoter activities under programmable logical operations.

## NATURAL AND SYNTHETIC GENE CIRCUITS

### Synthetic gene circuits and natural gene regulatory networks

Natural selection has sculpted gene regulatory networks that prioritise traits that enhance an organism's fitness over features that may be valued by human genetic engineers. While characteristics such as modularity, standardisation, and insulation of components of gene regulatory networks are considered advantageous when designing technologies to deliberately re‐engineer cellular processes, they have not been uniformly selected for in nature (Espinosa‐Soto, 2018). Many mutations lead to pleiotropic effects in an organism, where multiple distinct processes are disrupted when a single gene's function is altered (Ferreira Neres & Wright, 2024). Consequently, attempts to engineer natural gene regulatory networks can result in myriad unintended and undesired cellular changes. While the modification of natural gene regulatory networks has been taken advantage of during the selective breeding of crops (Sauvage et al., 2017), biotechnologists are increasingly attempting to modify traits through bottom‐up engineering approaches. This requires one to prioritise orthogonal approaches that minimise interference with natural cellular processes (Ferreira Neres & Wright, 2024). Therefore, synthetic gene circuits offer an alternative approach to customise gene expression patterns and introduce new plant traits while reducing interference with evolved cellular processes and risks of deleterious changes to natural plant cell functions.

**Figure 1:**
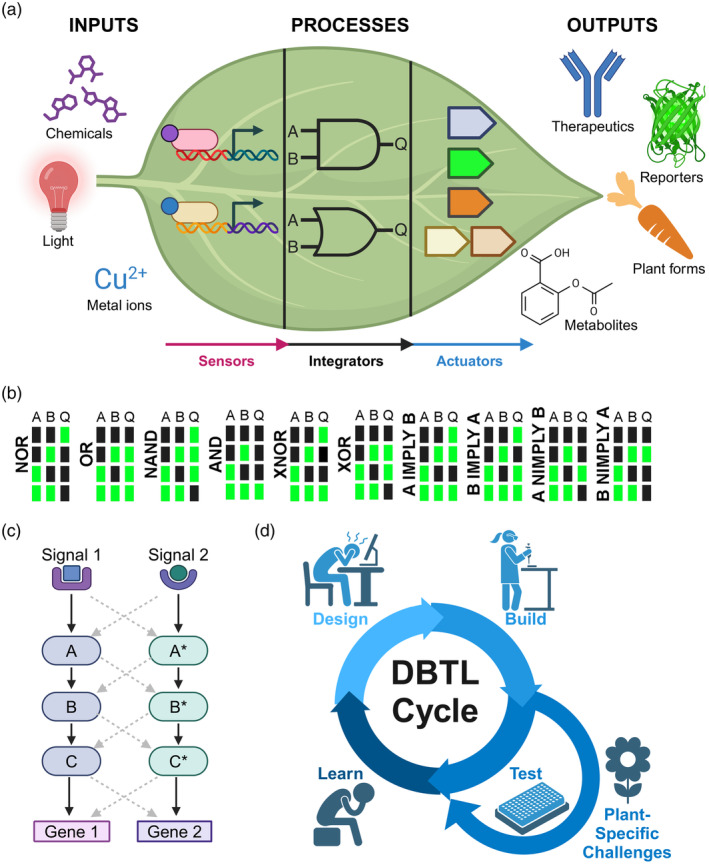
Synthetic gene circuits. (a) Synthetic gene circuit components can be divided up as sensors, integrators (processors) and actuators. Sensors convert non‐transcriptional signals (inputs, such as cell‐type or temperature) and convert them into a signal that alters the transcription of the integrator, which can process multiple input signals to produce an output based on the programmed Boolean logic. Pictured is the symbol for an AND gate (top) and an OR gate (bottom). The actuator converts the integrator's output into a plant response, such reporter protein expression for visualisation or production of useful chemical metabolites (example here is aspirin). (b) Truth tables for all of the two‐input Boolean logic gates. A and B represent the absence (black) or presence (green) of inputs 1 and 2, respectively. Q represents the activation (green) or repression (black) of the output. (c) Orthogonality is important to the functioning of synthetic gene circuits without interfering with natural processes within the plant. In this example, Signal 1 stimulates Gene 1 via a signaling pathway consisting of factors A, B, and C. In contrast, Signal 2 stimulates Gene 2 via an orthogonal signaling pathway consisting of A*, B*, and C*. The dashed grey arrows indicate a lack of cross‐reactivity between the two signalling pathways. (d) The Design‐Build‐Test‐Learn (DBTL) cycle is common in synthetic biology projects. With plants, the Test phase can be greatly prolonged by the long generation time of plants. Some figure components were created in Biorender.

Synthetic gene circuits utilise biological components (DNA, RNA and proteins) to sense and integrate input signals, producing an output that reflects a specific expression state based on programmed logical operations that compute the inputs. Broadly, synthetic gene circuits can be split into three core modules: sensors, integrators, and actuators (Jusiak et al., 2016) (Figure 1a). The sensor module acts to transmit input signals to the integrator (Figure 1a), which is the heart of the circuit and is sometimes known as processor (Vazquez‐Vilar et al., 2023; Xie et al., 2020). The integrator module receives the sensor signals and converts them into a message that the actuator module can perceive, often via a change in transcription. The integrator can be designed to sense multiple inputs and execute Boolean logic operations such as NOR, OR and AND, which dictate the combination of input signals that are required for the integrator and circuit to produce an output (Figure 1b). These operations thereby enable selective activation or repression of transgenes or endogenous genes and pathways. For instance, an AND gate would require the presence of two or more distinct input signals to activate a gene of interest, but not either signal alone (Figure 1b). This approach holds potential for applications such as inducing the production of a specific metabolite in a particular cell type or environmental condition when triggered by a human‐induced activation signal. Much recent progress in plant synthetic gene circuits has been made in the design, construction and testing of integrators (Anderson et al., 2023; Brophy et al., 2022; Ferreira & Antunes, 2024; Guiziou et al., 2023; Khan et al., 2024; Lloyd et al., 2022; Yang & Nemhauser, 2023). Finally, actuators are the output component(s) of the circuit, which could be transgenes to produce a reporter signal or desired product, or trans‐acting regulatory systems such as CRISPR/Cas‐based transcriptional regulators targeted to desired endogenous genes and pathways to alter their activity (Figure 1a). To date, simple reporters (e.g. GFP) have been the most commonly used actuators due to their value in testing circuit activity (Figure 1a).

#### Core concept—orthogonality

Orthogonality is a fundamental principle in the design of synthetic gene circuits, emphasising the use of genetic parts that strongly interact with each other but interact weakly with normal cellular components (Liu et al., 2018) (Figure 1c). This is usually achieved by using genetic components from other organisms (Calvache et al., 2024). For simple actuators, reporters like GFP and luciferases have been used in a range of organisms without an obvious penalty on plant growth and do not interfere with other cellular components. One example is the use of bacterial transcription factors (TFs) to construct synthetic gene circuits (Anderson et al., 2023; Brophy et al., 2022; Ferreira & Antunes, 2024). Unlike the endogenous plant TFs, which have binding sites within the genome and evolved functions, the use of bacterial TFs reduces interference with endogenous plant gene regulatory processes, thereby limiting the interactions of the synthetic gene circuit components with innate plant processes (Brophy et al., 2022). Similar principles apply to the use of recombinases from bacteriophage and yeast (Bernabé‐Orts et al., 2020; Guiziou et al., 2023; Lloyd et al., 2022; Maranas et al., 2024) or CRISPR/Cas‐based gene circuit platforms derived from bacteria (Khan et al., 2024; Yang & Nemhauser, 2023). While gene circuits must function within the cell, sharing the same pool of metabolites and competing for limited resources like RNA polymerases, reducing the level of cross‐talk with cellular processes is vital for the success and predictability of circuitry in living organisms. The long life cycles of plants also mean that the time it takes to test orthogonal components is longer than in microbes with short generation times (Figure 1d).

## THE ARCHITECTURE OF SYNTHETIC GENE CIRCUITS

When designing synthetic gene circuits, it is convenient to modularise the components into discrete units that each perform a specific function: sensing, computation and affecting a change in cellular output (Figure 1a). Below is an outline of each module and how they interact to create a full synthetic gene circuit.

### Sensors

Input signals used to drive synthetic circuits can come in many forms, from small molecules such as metabolites or drugs to cellular or developmental states, environmental conditions or specific wavelengths of light (Meyers, 2006; Ochoa‐Fernandez et al., 2020; Wang et al., 2013). Such internal or external cues that modulate the activity of specific endogenous or introduced plant promoters can be used to drive the input signals that are computed by a gene circuit (Bernabé‐Orts et al., 2020; Guiziou et al., 2023; Khan et al., 2024; Lloyd et al., 2022; Maranas et al., 2024; Yang & Nemhauser, 2023). Gene circuit systems commonly use such promoters to drive the expression of circuit components such as guide RNAs (gRNAs), recombinases, and sequence‐specific DNA binding proteins that mediate the molecular processes that form the circuit logic. In plants, a variety of naturally occurring or engineered inducible promoters have been used to respond to specific stimuli such as dexamethasone (Aoyama & Chua, 1997; Craft et al., 2005), β‐Estradiol (Zuo et al., 2000), ethanol (Zuo et al., 2000), metal ions such as copper (Garcia‐Perez et al., 2022, 2024), and environmental cues such as heat or light, which have been summarised recently (Wang & Demirer, 2023). Beyond transcription‐based sensors, other types of biosensors, including protein‐based fluorescent sensors, have also been developed for plant synthetic biology applications, broadening the range of potential inputs for gene circuits (Sadoine et al., 2021).

However, transcription‐based sensors relying on inducible promoters may lead to unintended or low‐level expression, or ‘leakiness’ (de Veylder et al., 2000), which is a significant limitation that can potentially compromise the performance and reliability of synthetic gene circuits driven by such inputs. Furthermore, a significant challenge with many engineered inducible promoters developed for plants, such as dexamethasone and the red/blue light‐controlled PULSE (Plant Usable Light‐Switch Elements) system (Aoyama & Chua, 1997; Craft et al., 2005; Ochoa‐Fernandez et al., 2020), is their highly repetitive nature. These repetitive sequences are inherently unstable in cells (Hossain et al., 2020) and could compromise the long‐term reliability and robustness of synthetic gene circuits in plants.

In addition to inducible promoters, cell‐type‐ and developmental‐stage‐specific promoters can be used to drive the input signals to feed into circuits (Marquès‐Bueno et al., 2016; Schürholz et al., 2018). For example, by using both inducible promoters and cell‐type‐specific or developmental promoters for producing gene circuit inputs, it is possible to reprogram gene expression in a spatiotemporally customised manner (Lloyd et al., 2022). However, such promoters reported to date are primarily limited to a few well‐studied species, in particular Arabidopsis. Application of single‐cell transcriptome and epigenome profiling technologies to a wider range of plant species and tissues will enable the discovery and construction of a diverse repository of such cell‐type‐ and condition‐specific regulatory regions that can be exploited as valuable components for building and controlling synthetic gene circuits (Marand et al., 2021).

A limitation in assessing the performance of synthetic gene circuits is the inability to quantify the activity of individual components, such as inputs or sensors, as their activity is typically assessed in conjunction with the overall circuit output. An example of this is the lack of CRISPRi circuit activity measured in stable transgenic lines after dexamethasone induction (Khan et al., 2024). This lack of repression was not due to a failure of the computational components of the circuit, but due to insufficient induction of expression of the circuit input gRNA by the dexamethasone signal (Khan et al., 2024). Poor induction of an input promoter in its on‐state is another potential failure point when designing and constructing circuits. For circuits that need to function in a plant exposed to the environment (dynamically), these risks could be mitigated by extensive characterisation of the input promoter dynamics to assess if it is appropriate for driving the circuit computation effectively.

### Integrators

**Figure 2:**
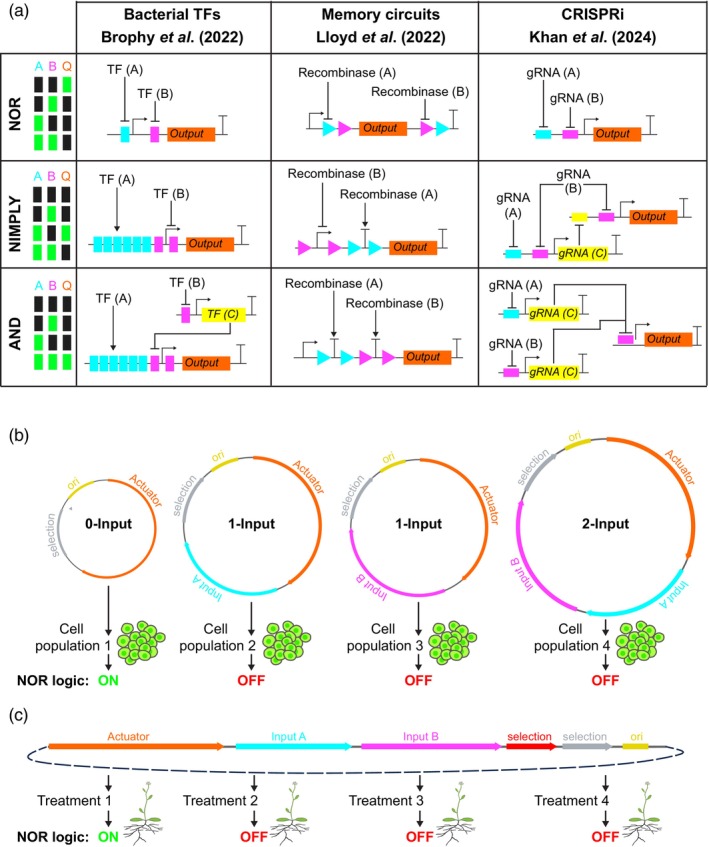
The diversity of synthetic gene circuit designs for plants. (a) Comparison of how NOR, NIMPLY and AND gate Boolean logic can be achieved with different synthetic gene circuit platforms: with bacterial TFs (Brophy et al., 2022), DNA recombinases (Lloyd et al., 2022) and CRISPRi (Khan et al., 2024), using the rearrangement of the same genetic components within each platform to achieve different computation. Input 1 and 2 are denoted by the letters A and B, respectively, with the output denoted as Q. Bacterial TFs can bind to and repress target promoter activity (T symbol) while others can activate the target promoter (arrow symbol). Recombinases act to remove DNA between recombination target sites (coloured triangles), removing the sequence between them and preventing function. Removal of a coding sequence prevents gene activity, while removal of a repressive sequence prevents repression. For CRISPRi circuits, the binding of dCas9 to a gRNA binding site within the promoter blocks expression. Activation is achieved through the repression of a repressor. (b) With a hardcoded circuit design, a plasmid representing a single static state is assembled and transfected into a population of cells. Different populations transfected with different plasmids (representing different states) are compared to determine whether the circuit design has performed as expected. (c) With a dynamic circuit design (soft coded circuit), a single plasmid is designed and transformed into a plant. Then different individuals are taken to undergo different treatment regimes to induce different inputs into the circuits. These could be environmental conditions such as temperature, light wavelength or light intensity. Tissue type may also be used as an inducing signal with developmental promoters.

In a synthetic gene circuit, the integrator module performs a logical operation to drive a specific transcriptional response based on the input signals received. Integrator units often consist of an engineered promoter or other regulatory sequence that contains customisable binding sites for input‐derived circuit components, such as DNA recombinases, sequence‐specific DNA binding proteins (TFs) and gRNAs/dCas9 systems that have been used in advanced plant circuits (Anderson et al., 2023; Brophy et al., 2022; Ferreira & Antunes, 2024; Guiziou et al., 2023; Khan et al., 2024; Lloyd et al., 2022; Yang & Nemhauser, 2023). These components interact with the integrator to alter its transcriptional output in a variety of ways. For example, DNA recombinases can be used to remove or invert activating or repressing sequences such as terminators, promoters or coding sequences (Guiziou et al., 2019; Weinberg et al., 2017). Sequence‐specific DNA binding proteins fused to transcriptional regulators can be recruited to customisable arrays of binding elements in an engineered promoter to alter its activity (Brophy et al., 2022). Furthermore, in a CRISPR interference (CRISPRi; Bikard et al., 2013; Qi et al., 2013) based circuit, the integrator promoter contains distinct customisable gRNA binding sites flanking the TATA box that can be bound by nuclease inactivated Cas9 (dCas9) alone (Khan et al., 2024) or dCas9 fused to a repressor domain (Khan et al., 2024; Yang & Nemhauser, 2023), leading to transcriptional repression (Figure 2a). Based on the configuration of these genetic elements, specific logical computations can be achieved. For example, a NOR gate can be achieved in a CRISPRi circuit in which an engineered integrator promoter has two different gRNA target sequences (Khan et al., 2024; Yang & Nemhauser, 2023), where the presence of either or both gRNAs will recruit dCas9 and repress transcription from the promoter (Figure 2a). The gene regulated by the integrator is often the output of the circuit (actuator, see below), but could also be an input to another downstream logic gate, creating a circuit made up of layered gates (Khan et al., 2024) (Figure 2a). Through such reconfiguration of a common set of modular genetic parts, any Boolean logic operation can be achieved (Figure 2a). With recombinases or bacterial TFs, changing the placement of binding sites around plant promoters can lead to different logical outcomes (Brophy et al., 2022; Lloyd et al., 2022), while with CRISPRi circuits (Khan et al., 2024), layering of multiple integrator promoters feeding into each other can achieve the desired circuit activity (Figure 2a), given that a NOR gate is a universal gate (Khan et al., 2024). Moreover, recombinases and bacterial TFs could also be layered in a similar manner to achieve complex logic functions. Modularity facilitates the construction of multi‐layered complex circuits, while the reconfigurability of these parts enables the connection of different input signals into circuits, which can then be interfaced with the host's endogenous pathways. Ideally, the components should also be compact and modular for easy stacking and assembly into circuits in a predictable manner and delivery to the host cell (Meyers, 2006; Wang et al., 2013). However, it is important to note that recombinases and bacterial TFs can also enable the direct implementation of certain logic circuits in a single layer (Brophy et al., 2022; Chen et al., 2020; Gaber et al., 2014; Lebar & Jerala, 2016; Lebar et al., 2014; Lloyd et al., 2022; Rantasalo et al., 2018; Weinberg et al., 2017), eliminating the need for NOR gate layering. In some cases, stacking NOR gates may introduce additional complexity and require more genetic components, making a single‐layer approach more practical for streamlined circuit design and integration (Khan et al., 2024).

The long testing cycles encountered when working with plants (Figure 1d), as well as the optimisation required to couple the sensor and integrator modules, have necessitated approaches for rapid testing of circuit computation in isolation, for example to avoid concurrent issues of input leakiness or lack of activation. Many researchers working have opted to characterise the circuit computation by omitting individual input factors from the DNA being transfected into cells (Brophy et al., 2022; Gaber et al., 2014; Khan et al., 2024; Lloyd et al., 2022; Weinberg et al., 2017). This effectively uses the presence/absence of DNA components encoding the input (sgRNA, TF, recombinase, etc.) to control the combination of input signals being tested (Figure 2b). In this approach, a single plasmid encodes a particular desired state (on or off) based on the input signals included in the plasmid, and separate populations of cells are transfected with different plasmids and compared to identify if the desired computation is achieved (Figure 2b). We describe this as a ‘hardcoded’ circuit design, as the circuit does not change in response to external factors after transfection (Figure 2b). In effect, the hardcoded circuit is static, with each input value forcibly set to 1 or 0, similar to how a computer program can be hardcoded to use values set within its own code, rather than expecting external input from a user. While this is a valuable strategy at the prototyping stage for iterative testing of integrator components, it has inherent limitations. Some circuit components, particularly those requiring dynamic regulation in response to external signals, cannot be fully characterised or optimised in a hardcoded framework. For example, circuits integrating environmental or developmental cues must be evaluated in a ‘dynamic’ circuit design, where a single plasmid encodes a circuit capable of switching between on and off states based on external stimuli (chemical, temperature, or tissue specificity; Figure 2c). This is analogous to a computer program that is soft coded and awaits inputs from the user in order to change the output. Unlike the binary nature of hardcoded circuits, inputs in dynamic circuit designs can take continuous values rather than discrete on/off states. In natural biological systems, the threshold for activation is not fixed but instead depends on multiple factors such as cell type, developmental stage, cell health and environmental conditions. These additional variables necessitate experimental validation to determine how an input signal dynamically regulates circuit function.

Dynamic circuit characterisation is crucial for fine‐tuning sensor sensitivity, integrator thresholds and actuator performance in real biological systems (Brophy et al., 2022; Khan et al., 2024; Lloyd et al., 2022). To overcome the challenges of simultaneous testing of sensors, integrators and actuators, a simple approach focuses on optimisation of a component of interest, such as an integrator. By using a simple, well characterised reporter as an actuator, the input can be simulated by the presence or absence of the encoding DNA, enabling direct testing of an integrator in a controlled, predefined manner (Hardcoded circuit; Figure 2b). This is a valuable strategy at the prototyping stage to expedite iterative testing of a robust set of integrators and has been widely used in plants through the use of protoplasts or tobacco transient leaf infiltrations (Brophy et al., 2022; Khan et al., 2024; Lloyd et al., 2022). However, circuits optimised in a hardcoded design may not function identically when implemented in a dynamic framework, as unexpected genetic interactions, chromatin accessibility and cellular heterogeneity can alter circuit behaviour. This is a challenge not only in plant systems but also in other systems, where factors such as epigenetic modifications and transcriptional noise can affect circuit function (Keung et al., 2015; Murphy et al., 2010). Note that even in a hardcoded circuit, it may take time for the steady‐state activity to be reached (Lloyd et al., 2022). For circuits intended to function dynamically within plants, transitioning from hardcoded to dynamic circuit characterisation is essential to capture the full complexity of regulatory interactions *in vivo* (Brophy et al., 2022; Khan et al., 2024; Lloyd et al., 2022).

Beyond the more commonly used gene circuit platforms, bacterial allosteric transcription factors (aTFs) present a promising tool for constructing synthetic gene circuits. These regulatory proteins bind to specific DNA sequences, and their activity is modulated by the presence of ligands or metabolites (Li et al., 2023). Ferreira and Antunes (2024) demonstrated the utility of bacterial aTFs in plants, using them as biosensors responsive to phenylpropanoid‐related metabolites. Notably, while this study highlights the feasibility of aTF‐based sensing in plants, only one out of the five tested aTFs (TtgR) successfully responded to its target ligand. This underscores key challenges, including constraints imposed by promoter context, limited nuclear accessibility of certain ligands, and potential metabolite compartmentalisation within enzyme complexes. These factors may hinder aTF performance in plants and necessitate further optimisation, such as redesigning synthetic promoters, refining response element positioning and enhancing aTF‐ligand interactions. Nevertheless, this approach fuses the sensor and integrator modules (Figure 1a), and highlights the potential of aTF‐based circuits to provide dynamic and precise control over plant metabolic pathways, paving the way for advancements in biosynthetic engineering and the development of traits linked to metabolic regulation (Ferreira & Antunes, 2024).

### Actuators

The actuators of synthetic gene circuits are used for transmitting the output signal from the integrator to alter cell function (Jusiak et al., 2016), for example targeting endogenous genes to alter their expression. Reporter genes are particularly useful as actuators for circuit development and optimisation because they can be detected visually and can be quantified, enabling characterisation of circuit performance, as used in most gene circuit design studies (Anderson et al., 2023; Brophy et al., 2022; Ferreira & Antunes, 2024; Guiziou et al., 2023; Khan et al., 2024; Lloyd et al., 2022; Yang & Nemhauser, 2023). Once the circuit is optimised to a desired level, the reporter genes can be replaced with genes of interest to alter specific cellular functions (Khakhar et al., 2018; Meyers, 2006; Wang et al., 2013). For example, Brophy et al. used *solitary root* (*slr‐1*) as an actuator in a one‐input switch (YES or BUFFER gate, which outputs the same on/off state as it receives) to control root branch density in Arabidopsis (Brophy et al., 2022), demonstrating that circuits can be coupled to actuators in order to alter plant form (Figure 1a). In the future, more radical changes to plant form and function could be achieved with the spatiotemporal patterning that can be achieved with synthetic gene circuits, such as altering shoot branching number or angle, or leaf shape. Some of these changes will require multiple input signals to control cell fate decisions and the biochemical pathways expressed in each. With further refinement of synthetic gene circuits, they could be a useful tool in ambitious projects, such as the conversion of C3 crops to use C4 photosynthesis (Furbank et al., 2023) or to engineer nitrogen fixation into cereals (Jhu & Oldroyd, 2023). For example, the C4 rice project has designed modified rice strains that include the use of synthetic promoters for multiplexing cell‐type specificity of target genes (Danila et al., 2022; Furbank et al., 2023) and could benefit from further conditional control of expression through the use of logic gate circuits. Synthetic gene circuits can also be used to express desired protein products, such as therapeutic proteins directly or they can control the expression of an enzyme that catalyses the synthesis of a valuable metabolite of interest (Figure 1a; Anderson et al., 2023; Brophy et al., 2022; Ferreira & Antunes, 2024; Guiziou et al., 2023; Khan et al., 2024; Lloyd et al., 2022; Yang & Nemhauser, 2023).

DNA binding proteins, including endogenous TFs or CRISPR/Cas systems such as CRISPR activation (CRISPRa) or CRISPRi, can also be employed as actuators to regulate endogenous gene networks and alter critical traits such as stress resistance, growth patterns and metabolic output in plants. Beyond TFs, key metabolic enzymes, phosphorylation cascades, chromatin remodelers and defence proteins could serve as actuators in synthetic circuits, mediating diverse cellular functions. Similar approaches have been explored in bacterial systems, where CRISPR‐based synthetic circuits have been used to regulate gene expression and cellular behaviors (Brophy & Voigt, 2014; Nielsen & Voigt, 2014). The selection of an appropriate actuator is determined by the desired circuit outcome and the specific biological or metabolic pathway targeted for modulation. This diversity highlights the broad range of what may be considered actuators in synthetic gene circuits in order to enable desired control of cellular processes and plant trait introduction.

## CHALLENGES IN BUILDING AND TESTING CIRCUITS

Achieving robust and correct function of a circuit composed of many different genetic parts is like conducting an orchestra, where the suboptimal performance of a single part can ruin the whole effort. Therefore, when designing any circuit, the simplest possible configuration should be attempted first to reduce the chances of unexpected genetic interactions leading to suboptimal performance. This relates to the engineering KISS principle: Keep It Simple (Stupid), avoiding needless over‐engineering of a design. Beyond this, there are a number of challenges that plant biotechnologists need to overcome when designing and implementing synthetic gene circuits.

### Core concept—design‐build‐test‐learn (DBTL) cycles

The design‐build‐test‐learn cycle is core to engineering biological systems (Opgenorth et al., 2019) (Figure 1d). The development of the first synthetic gene circuits was achieved in bacteria (Elowitz & Leibler, 2000; Gardner et al., 2000). Bacteria like *E. coli* are the favorite synthetic biology chassis because they divide so rapidly, such that a single bacterium containing a circuit‐encoding plasmid can be expanded to a population of millions in hours. This population can then be subdivided and exposed to different conditions to measure circuit activity. Yeast and mammalian cells do not divide as quickly as *E. coli* but are still fast‐growing and readily modified *in vitro*, and therefore have been used to develop many circuit designs in eukaryotes (Chen et al., 2020; Gaber et al., 2014; Lebar et al., 2014; Lebar & Jerala, 2016; Rantasalo et al., 2018; Weinberg et al., 2017). However, when working with stably transformed whole plants, the Build/Test phases can be very long (Figure 1d). Even for plants with relatively short life cycles, like Arabidopsis, obtaining plants homozygous for a construct inserted into the genome can take many months. With crops that grow even slower, such as maize, this process can take years (Yassitepe et al., 2021). For plants with an even longer generation time, such as trees, the timeline is extremely long. Furthermore, stable insertion of constructs into plant genomes is very challenging for most species, often involving inefficient delivery into de‐differentiated plant callus followed by lengthy regeneration times, and is encumbered by extremely low efficiency of targeted gene insertion or chromosomal position effect variation of randomly inserted constructs that necessitates laborious downstream screening of many independent transformant lines (Singer et al., 2012). Recent advances in targeted gene insertion provide enticing new pathways to reduce the challenges of precise transgene insertion (Liu et al., 2024; Sun et al., 2024); however, a variety of other processes present ongoing challenges to the rapid and high‐throughput generation of modified plant lines. The design and build phase (DNA assembly), as well as the learn phase, will be a similar length of time regardless of organism, but for many plants, the test phase becomes greatly extended compared to organisms that can be cultured easily in the lab (Figure 1d). This significantly slows the rate of progress for plant synthetic biologists and is a contributing factor in progress in plant gene circuit development lagging behind that of other kingdoms. To circumvent the long generation times of plants, many researchers have used transient expression systems, such as isolated plant cells (protoplasts) or tobacco leaf infiltration systems (Brophy et al., 2022; Khan et al., 2024; Lloyd et al., 2022) to accelerate the test phase. Circuit designs can be rapidly assembled and tested in protoplasts or tobacco leaves, issues with the design can be identified, and the DBTL cycle can be used to rapidly cycle through multiple designs before then testing a design in stable transgenic plants. This approach mitigates risks and reduces the overall development time of synthetic gene circuits, though challenges remain in differences between the constructs delivered in these transient systems compared to the final objective of a stably integrated construct in the genome, as well as testing being restricted to only certain cell and tissue types that may differ from the contexts for which the circuits are ultimately being developed. Ultimately, functionality must be tested in stable transgenic plants.

Despite efforts to accelerate the test phase using transient expression systems, achieving functional gene circuits still requires multiple iterations of the DTBL cycle (Opgenorth et al., 2019). For example, a GFP output gene used in tobacco leaves needed an intron added to prevent bacterial expression of the output gene from being detected in the plant leaves (Brophy et al., 2022), and introns were added to recombinase genes to prevent premature recombination in bacteria from leaky expression (Lloyd et al., 2022). Guiziou et al. (2023) developed recombinase‐based memory circuits that switched under the control of a lateral root‐induced promoter; however, multiple designs were tested to find the best‐performing circuit (Guiziou et al., 2023). Varying the sensor (lateral root specific promoter) greatly altered the non‐lateral root switching of the circuit, but by varying recombinase activity, the specificity of switching could be improved (Guiziou et al., 2023). Such design changes are needed for the optimal circuit performance to be achieved, but the long time it takes to engineer plants limits this.

### Core concept—rational design of the irrational

Natural selection often does not select for what a human engineer would consider the most logically designed system. Evolution fixes genetic changes within a population based on a reproductive advantage at the time, regardless of how modular and upgradable the system is in the future. This unguided design produces a functional but complex biological machine that lacks easy modes of expansion and upgradability. Unlike pure engineering approaches, we cannot simply predict the local behaviour of a cellular component or process based on first principles (or the laws of physics). For example, the activity of a promoter sequence used to induce transcription in one species will likely function distinctly in a different species. Moving genetic parts between species is akin to moving a computer program between operating systems, where there would be no expectation of it working without major initial incompatibility issues. While we can attempt to rationally design robustly performing synthetic gene circuits, we are limited by what we can achieve from rational design alone. In protein engineering, rational design can help guide a change in protein function, but rounds of *in vitro* selection to identify random variants with improved function can be effective in identifying solutions (Herud‐Sikimić et al., 2021). Given the complexity of biological systems and the artificial nature of gene circuit technologies, we still lack the ability to fully rationally design modifications of many biological processes without relying on selective processes to guide or refine this process. An *in vitro* evolutionary selection approach or very high‐throughput construct synthesis and quantitative functional assessment provide avenues to explore a very large parameter space of circuit designs to identify those that achieve the desired behaviour. When coupled with computational learning approaches, this has the potential to reveal underlying design features that robustly produce the desired circuit activities.

While these high‐throughput approaches offer powerful ways to optimise circuit designs, several key challenges remain in implementing synthetic gene circuits in practice. The sequence context dependency of regulatory regions can cause significant challenges during synthetic gene circuit DBTL cycles and requires careful assessment of activity (Stone et al., 2024). Construct architecture can also have a significant impact on circuit performance, as demonstrated by unexpected changes caused by altering the plasmid backbone in bacteria (Tas et al., 2020) and unintended read‐through of transcription between transcriptional units in plant CRISPRi circuits that were resolved by additional terminator engineering (Khan et al., 2024). The stability of synthetic gene circuit performance over multiple generations is also of vital importance, as being able to maintain desired circuit performance in crops in the field is essential for future implementation of this technology in agriculture. This is particularly important for memory circuits that rely upon stable genetic changes to the circuit DNA sequence to alter function (Bernabé‐Orts et al., 2020; Guiziou et al., 2023; Lloyd et al., 2022; Maranas et al., 2024), where premature recombination from a leaky sensor module that leads to recombinase expression before the intended trigger could break the whole design. Guiziou et al. (2023) studied the stability of various memory circuit designs over multiple generations of Arabidopsis plants, finding that the stability varied depending on which sensor module was used (Guiziou et al., 2023) and highlighting the importance of careful circuit input selection and testing. Recombinase activity is also important in determining memory circuit stability over multiple generations, where pairing a leaky sensor module to a low‐activity recombinase can help to reduce premature recombination (Guiziou et al., 2023). Engineering biological systems poses unique challenges, but great progress has been made and paves a path for the application of synthetic gene circuits in agriculture.

## CURRENT CHALLENGES AND FUTURE DIRECTIONS OF SYNTHETIC GENE CIRCUITS IN AGRICULTURE AND RESEARCH

Despite the aforementioned advances in plant gene circuits in recent years, the adoption of synthetic biology approaches in plants has been slower than in microbial and mammalian systems due to a variety of challenges. Unlike microbial systems, which benefit from model organisms such as *E. coli* and *S. cerevisiae*, or mammalian systems with widely used stable cell lines and differentiation capabilities, plant researchers lack a comparable range of stable cell lines with defined identities that are simple to propagate and undergo single‐cell selection. The absence of plant equivalents to lentiviral and other viral‐based DNA delivery systems prevents efficient, stable integration of genetic constructs at controlled multiplicities of infection, as well as their subsequent selection and propagation. This limitation hinders the delivery of single genetic constructs to individual cells, a fundamental requirement for large‐scale pooled strategies in mammalian systems that enable parallelized construct screening. The extremely low efficiency of gene targeting in most plant species leads to largely random construct insertion, requiring extensive screening of independent transformants to mitigate position‐dependent effects on construct performance. Furthermore, regeneration of whole plants and multi‐generational propagation to isolate homozygous plants is typically required to obtain uniformly modified cell populations. The implementation of circuits in animal cell lines rarely involves demonstration of the function of these circuits in whole multicellular organisms (Gaber et al., 2014; Lebar et al., 2014; Weinberg et al., 2017); meanwhile, this has often been the case for circuits implemented in plants (Brophy et al., 2022; Guiziou et al., 2023; Khan et al., 2024; Lloyd et al., 2022). Cell walls in plants complicate transformation and gene delivery, as well as individual cell selection. While enzymatic digestion to generate protoplasts is commonly used, this process can be damaging to cells. These challenges, amongst others, significantly impede the ability to rapidly prototype and test synthetic constructs in plants.

This discrepancy between plant and mammalian systems also highlights the need for better predictive tools and computational models to bridge the gap between transient expression data or cell culture experiments and whole‐plant behaviour. Importantly, such models must incorporate cell‐type‐specific contexts, as gene circuit performance can vary depending on developmental stage, metabolic state and environmental cues. Developing these tools, alongside standardised protocols, could streamline validation processes, minimize dependence on whole‐plant regeneration and improve comparability across plant and mammalian systems.

Despite these challenges, plant synthetic biology offers tremendous opportunities for innovation in agriculture, bio‐based production and environmental sustainability. Advances in genome editing, synthetic promoters and modular genetic parts are paving the way for more efficient and precise plant engineering. Furthermore, the integration of computational models and high‐throughput screening platforms could help overcome technical barriers and accelerate the design‐build‐test‐learn cycle. By addressing these challenges and leveraging insights from microbial and mammalian systems, the plant synthetic biology community can unlock its full potential and drive transformative advancements in the field.

With the recent development of multiple plant synthetic gene circuit platforms, biotechnologists have a range of molecular tool options for developing trait‐conferring circuits in crops. The choice between distinct operating principles, for example a memory circuit using recombinases (Bernabé‐Orts et al., 2020; Guiziou et al., 2023; Lloyd et al., 2022; Maranas et al., 2024) or reversible circuits using transcriptional control (Anderson et al., 2023; Brophy et al., 2022; Ferreira & Antunes, 2024; Khan et al., 2024; Yang & Nemhauser, 2023), is important and will depend on the application and trait that is being pursued. For example, reversible expression may be appropriate for factors that confer resilience against transient environmental stresses, while it may be preferred to irreversibly trigger expression of a valuable biomolecule once sufficient plant biomass is achieved, to maximise production and limit effects on growth. Synthetic gene circuits have the potential to introduce a range of traits to solve various problems in agriculture, from abiotic stress or pathogen protection to bioproduction of useful compounds. Currently, the molecular tools to define when and where a gene is expressed are very limited in plants and can cause unintended side effects through pleiotropy (Ferreira Neres & Wright, 2024). With rational design of gene expression patterns, novel traits not otherwise possible can be engineered into crops. For example, a synthetic gene circuit could be constructed to sense the local soil nutrient conditions and confer the most ideal root system architecture. When coupled with development in more advanced synthetic sensor modules, this could allow for human‐timed induction of traits to coincide with forecasted weather events to protect crops. Not all traits will be useful in all plants; therefore, the choice of the host plant will be very important and will be influenced by the choice of circuit design and the application. One important consideration is whether the host organism will be suitable for the genetic tools needed for the synthetic gene circuit to function. The problem of host differences is not specific to plants—even in bacteria, the host organism's strain for a simple circuit has a large impact on circuit behaviour (Tas et al., 2020). This variation determined by the host could also be seen as a strength by testing different circuit designs in various hosts to find an optimal design (Tas et al., 2020). Monocots and dicots diverged ~200 million years ago (Wolfe et al., 1989), and many genetic parts do not behave the same between these two groups of plants (Gorjifard et al., 2024; Horstmann et al., 2004; Jores et al., 2021; Khan et al., 2024). Genetic parts that are widely applicable between a wide range of species will be useful, but species‐specific parts, such as promoters to act as sensor modules, are critical for circuit functionality. However, currently, the knowledge and availability of such components are extremely limited, highlighting the need to identify novel species‐specific genetic parts for developing synthetic gene circuits in crops. High‐throughput assays such as STARR‐seq (Self‐Transcribing Active Regulatory Region sequencing) are able to test multiple genetic parts in plant cells at once, allowing for rapid testing of some component types and will be valuable for expanding synthetic gene circuit technologies (Gorjifard et al., 2024; Jores et al., 2021). Advances in single‐cell transcriptomics, chromatin accessibility profiling, and spatial transcriptomics in crops are enabling the discovery of cell‐ and species‐specific regulatory elements (He et al., 2024; Long et al., 2024; Marand et al., 2021), which will facilitate the implementation of synthetic gene circuits in diverse plants through scalable discovery of novel regulatory elements needed as inputs. Furthermore, the implementation of new technologies for targeted transgene insertion in flowering plants (Liu et al., 2024; Sun et al., 2024), such as the transposase‐assisted target‐site integration method (Liu et al., 2024), will be critical to reducing the work required to screen through many transgenics caused by insertion position variation. By enabling site‐specific integration of genetic elements, this approach minimises off‐target effects and genome disruption, ensuring more reliable and predictable circuit functionality. Such a high rate of gene targeting is already possible in the moss *Physcomitrium* (*Physcomitrella*) *patens* (Chen et al., 2024; Kamisugi et al., 2005) and could be leveraged for the development of synthetic gene circuits in a land plant without the high level of variation due to chromosomal position variation of flowering plants (Singer et al., 2012).

Host‐aware circuit designs may help alleviate some of these challenges by building into circuit designs the sensing of limited host resources and have been pioneered in microbial synthetic gene circuits (Stone et al., 2024). Emergent properties can arise when synthetic gene circuits have unpredictable interactions with the host biology and can negatively impact host growth, creating feedback to prevent these negative outcomes by limiting circuit activity when it is a burden on the host (Stone et al., 2024). This problem of unpredicted emergent properties stems from the synthetic components not being truly orthogonal (Figure 1b) and instead having undesired effects on the host. Less reliance on host biochemical pathways would reduce unintended outcomes but greatly adds to the time needed to optimise a synthetic gene circuit through DBTL cycles. Competition over ribosomes for translation (common issue in bacterial circuits) or competition over RNA polymerase for transcription (common in mammalian circuits) is a particularly tricky problem to face, as all of these processes depend on the host machinery, which represents a finite resource within the cell (Stone et al., 2024). Growth feedback can fix this (Stone et al., 2024), but such a challenge is daunting given our limited knowledge of plant synthetic gene circuits. However, mathematical modeling of synthetic gene circuits in plants has the potential to increase our predictive power and thus reduce the number of DBTL cycles needed in order to achieve the desired circuit performance. Plant synthetic biology has rarely embraced mathematical modelling of circuits or circuit components (Kong et al., 2025), but this is widespread in the circuit design of other organisms (Bowyer et al., 2020; Ceroni et al., 2010; Espah Borujeni et al., 2020; Huang et al., 2018; Merzbacher & Oyarzún, 2023; Park et al., 2020). However, given the long generation time of plants, collecting data to characterize circuits and create a predictive model is a significant obstacle, but it will be vital for the long‐term success of plant synthetic gene circuits. Computational predictions of synthetic promoters in plants have proven to be accurate and indicate the potential modeling has for plant synthetic biology (Cai et al., 2020).

Once the challenges with the development of synthetic gene circuits in a range of plant species have been overcome, their potential to enable the introduction of radically new traits into crops that rely upon the integration of multiple sources of information, such as the environment, developmental stage and biotic challenges, will be fully realised. Such a dynamic response would be hard or impossible to introduce by traditional breeding or transgenic approaches. Furthermore, synthetic gene circuits can enhance basic plant research by offering new genetic tools for more precise modification of spatiotemporal expression patterns to understand basic plant processes. Mutant collections have already allowed researchers to understand a range of interesting processes (O'Malley & Ecker, 2010), however, the mutation is present in all cells of the organism, making lethality an issue in some cases, and complex phenotypes in which multiple cell types interact are difficult to interpret. With synthetic gene circuits, multi‐conditional activation or repression of target genes could be achieved (Watson et al., 2024). Synthetic tools are already being used to reveal new cell fate processes in plants with the application of memory circuits for detailed and complex lineage tracing (Maranas et al., 2024). Through the process of engineering gene circuits, challenges will likely also reveal the unknowns of how plant gene expression pathways operate and reveal new biological mechanisms, similar to how early GM plants informed our understanding of co‐suppression (Napoli et al., 1990; van der Krol et al., 1990) and underpinned discoveries in RNA interference and RNA‐directed DNA methylation (Rajeevkumar et al., 2015). By troubleshooting gene circuits, we can uncover new principles that govern the biology that we are trying to manipulate. This is reminiscent of the Richard Feynman quote “What I cannot create, I do not understand” (Alberts, 2011).

## SUMMARY AND OUTLOOK

Synthetic gene circuits have the potential to greatly expand the traits that we can engineer into plants by integration of multiple stimuli to fine‐tune the expression of introduced transgenes or endogenous pathways. However, a variety of technical hurdles must be overcome to implement synthetic gene circuits in agriculture. Biotechnologists need to continue their characterisation of genetic parts across a wide range of species to determine which perform appropriately across different cellular contexts (different local rules of the cell). Moreover, it is also important to ensure that all genetic parts within a circuit work together effectively. With the extremely long transformation and generation times of plants, especially some crops (Figure 1d), this makes plants a particularly challenging system to engineer. Despite this, GM traits have a long history in plant agriculture (Raman, 2017) and even in conservation efforts, such as with the American Chestnut (Powell et al., 2019). While some parts of the world are still reluctant to grow GM crops (Kovak et al., 2022), other parts of the world have embraced them and grow them on large areas of land (Raman, 2017). As climate change threatens global food security over the coming decades (Archibald et al., 2023), we may see a change in public perception of GM crops as the need for the unique benefits they can deliver becomes more apparent. Therefore, it is important that the translation of synthetic gene circuits to application starts now, so that these technical challenges can be addressed early and a sufficient level of technological maturation is achieved in time to deliver solutions when required (Boxes 1 and 2).

**Key points**

- Synthetic gene circuits enable sophisticated control over plant traits by integrating multiple input signals through programmable logical operations that determine gene expression patterns.
- Circuit components can be divided into three main categories: sensors that detect input signals, integrators that compute the response, and actuators that produce the desired output.
- Orthogonality is crucial for circuit function, where components should interact strongly with each other but minimally with host cellular processes to avoid unintended effects.
- The long generation times of plants significantly extend the Design‐Build‐Test‐Learn cycle compared to other organisms, necessitating efficient prototyping strategies like transient expression systems.
- Recent advances in circuit platforms including bacterial transcription factors, recombinases and CRISPR systems provide diverse tools for engineering plant traits.

**Open questions**

- How can we accelerate the identification and characterisation of species‐specific genetic parts, particularly promoters, to enable circuit function across diverse crop species?
- What strategies can improve the predictability of circuit behaviour when moving from prototyping systems to stable transgenic plants?
- How can mathematical modeling be effectively applied to plant synthetic gene circuits to reduce the number of design iterations needed?

## AUTHOR CONTRIBUTIONS

All authors conceived, wrote and edited the manuscript. JPBL prepared the figures, with additions from AK.

## CONFLICT OF INTEREST

The authors declare that they have no competing interests.

## Data Availability

Data sharing is not applicable to this article as no new data were created or analyzed in this study.
